# Probing Changes in the Local Structure of Active Bimetallic Mn/Ru Oxides during Oxygen Evolution

**DOI:** 10.1021/acsaem.3c01585

**Published:** 2023-08-16

**Authors:** Michelle P. Browne, Carlota Domínguez, Can Kaplan, Michael E. G. Lyons, Emiliano Fonda, Paula E. Colavita

**Affiliations:** † School of Chemistry, CRANN and AMBER Research Centres, Trinity College Dublin, College Green, Dublin D02 PN40, Ireland; ‡ Helmholtz Young Investigator Group Electrocatalysis: Synthesis to Devices, Helmholtz-Zentrum Berlin für Materialien und Energie, 14109 Berlin, Germany; § SAMBA Beamline, SOLEIL Synchrotron, L′Orme des Merisiers, Saint-Aubin, BP48, 91192 Gif-sur-Yvette, France

**Keywords:** X-ray absorption spectroscopy, oxygen evolution reaction, operando, mixed oxides, water splitting

## Abstract

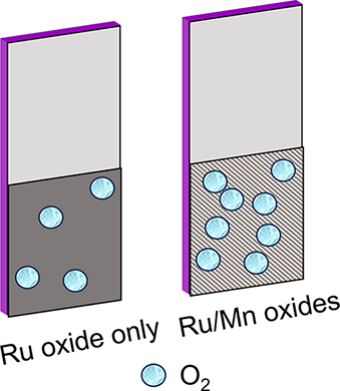

Identifying the active site of catalysts for the oxygen evolution reaction (OER) is critical for the design of electrode materials that will outperform the current, expensive state-of-the-art catalyst, RuO_2_. Previous work shows that mixed Mn/Ru oxides show comparable performances in the OER, while reducing reliance on this expensive and scarce Pt-group metal. Herein, X-ray photoelectron spectroscopy and X-ray absorption spectroscopy (XAS) are performed on mixed Mn/Ru oxide materials for the OER to understand structural and chemical changes at both metal sites during oxygen evolution. The results show that the Mn-content affects both the oxidation state and local coordination environment of Ru sites. Operando XAS experiments suggest that the presence of MnO_*x*_ might be essential to achieve high activity likely by facilitating changes in the O-coordination sphere of Ru centers.

## 1 Introduction

Electrolytic water splitting is an attractive process for producing clean hydrogen gas from renewable sources. Hydrogen gas can be used as an energy carrier to be stored or utilized in a fuel cell to generate electricity on demand, which is the basis of the future hydrogen economy concept.^1,2^ Unfortunately, electrolytic water splitting is still in the research and development stage as the catalysts currently used are expensive and scarce, thus preventing cost-competitive generation of green hydrogen at scale.^3^ The reaction of interest in the overall electrolytic water splitting process is the reaction that takes place at the cathode, the hydrogen evolution reaction (HER), as this is the electrode where H_2_ is produced.^4,5^ However, the bottleneck of the water splitting process is the reaction that takes place at the anode, the oxygen evolution reaction (OER), that yields O_2_ as a product.^6−10^ The optimum electrocatalysts for the OER are IrO_2_ and RuO_2_, both of which are Pt-group metals (PGMs) which are expensive and scarce.^11,12^ Large-scale utilization of water splitting will therefore require the development of new electrocatalysts with similar activity to the aforementioned PGMs but that are less costly and more earth abundant.^13^ The development and design of new, sustainable, and active materials as catalysts for the OER is therefore an important step toward enabling electrolytic water splitting.

In recent years, various reports have emerged on the high activity of mixed Mn/Ru-based oxide catalysts for the OER.^14−17^ For example, Pascuzzi et al. have reported on the effect caused by the addition of various amounts of Mn to RuO_2_-TiO_2_ on the OER when compared to pure RuO_2_-TiO_2_. In the study by Pascuzzi et al., a catalyst containing 44% Mn was the optimum OER catalyst which was reportedly due to a higher electrochemical surface compared to the pure RuO_2_-TiO_2_ area due to the insertion of the Mn into the Ru lattice.^14^ Additionally, through first-principles calculations, Lin et al. showed that the excellent performance of a Ru/MnO_2_ OER catalyst was due to a reduced energy barrier mechanism only involving *O and *OH species as intermediates.^18^

Furthermore, we have previously reported on a range of highly active OER catalysts based on mixed Mn/Ru oxides fabricated from thermal decomposition of precursor salts at the annealing temperature of 350 °C.^15^ These catalysts displayed excellent OER activity despite containing significantly lower Ru concentrations in the oxide catalyst matrix than pure RuO_2_. Ex situ XRD and FTIR measurements revealed that Mn centers in these high-performing materials possessed a mixed Mn^2+^/Mn^3+^ oxidation state, while Ru was in the +4 oxidation state in the as-synthesized material. However, the oxidation states and local structures of the metal centers during or after the OER were not investigated, despite these changes being important for an understanding of the active sites in these mixed Mn/Ru oxides and for future design and optimization of alternative sustainable mixed oxide electrocatalysts.

During the last decade, ex situ and in operando X-ray absorption spectroscopy (XAS) has been successfully used as a tool for investigating the active sites of various metal oxides as OER catalysts, including pure Mn oxide and Ru oxides.^19−24^ XAS is particularly useful due to its sensitivity to the local structure of metal centers including those embedded in amorphous or disordered phases that might play important roles in determining OER activity, but that are not amenable to XRD characterization. For example, Jaramillo and co-workers have extensively employed XAS for the characterization of MnO_*x*_ catalysts to investigate various parameters affecting their OER activity, such as applied potential, porosity, and the role of the support.^19,20,25^ Lian et al. have also characterized porous solvothermally prepared MnO_*x*_ materials under various annealing temperatures by XAS.^21^ The authors concluded that the annealing temperature plays an important role in determining the local structure and the Mn oxidation states of the prepared MnO_*x*_ catalysts, which in turn relates to activity trends in the OER. Additionally, XAS has been extensively utilized to determine the structure–activity relationships for pure and mixed RuO_2_ heterovalent substituted materials based on Fe, Ni, Co, Zn, and Ir. These studies revealed the active site of RuO_2_-based materials to be the two penta-coordinated transition metal cations in the rutile structure with a bonding distance from the central Ru atoms of 3 Å.^22,26,27^ The change in the RuO_2_ local structure resulting from the heterovalent substitutions alters the OER performance of the mixed material compared to the pure RuO_2_.

Notably, to the best of our knowledge, there have been no in situ/operando studies that investigate the local structure in OER active mixed Mn/Ru oxide materials despite these being excellent candidates for the fabrication of low-cost OER electrocatalysts. In this study, we aim to address this gap and characterize the local chemistry and structure of thermally prepared mixed and pure Mn/Ru oxides ex situ and in operando via XAS experiments to establish changes in the oxides before and after the OER. This will enable a new understanding of the origins of activity and develop design principles for novel mixed-oxide low-cost catalysts.

## 2 Experimental Methods

### 2.1 Electrode Fabrication

Pure and mixed Mn/Ru oxide electrocatalysts were prepared on Ti-coated Si wafers; a 150 nm thick Ti layer over a 50 nm Au layer was deposited onto the clean wafers using a Temescal FC-2000 electron beam evaporation system. Ti was chosen as a conductive support because it does not display OER activity over the potential window investigated.^15^ Five 0.2 M precursor solutions were made by dissolving (CH_3_COO)_2_Mn·4H_2_O and RuCl_3_·*x*H_2_O in butanol in different ratios; solutions were prepared in separate 10 mL conical flasks and then were evaporated on a hot plate until minimal solvent remained, thus forming the precursor pastes used to prepare the working electrodes. A coat of the relevant metal paste was applied to Ti/Si substrates which covered an area of 1 cm^2^, followed by drying in an oven at 90 °C for 10 min, and this process was repeated once. The resulting electrode was annealed in air for 9 h at 350 °C to ensure the decomposition of the precursor materials. This yielded Ti-supported oxide film electrodes of thickness 0.24 ± 0.6 μm, as estimated by profilometry. Samples are indicated by the % molar content of Mn in the mixed Mn/Ru precursor slurries; e.g., Mn 100 indicates a sample prepared from the 100% Mn precursor and treated at 350 °C in air.

### 2.2 Characterization Methods

X-ray photoelectron spectroscopy (XPS) measurements reported were taken using a VG Scientific ESCALab MKII system using an Al Kα X-ray source (1486.7 eV). The pass energy was set at 200 and 20 eV for the survey and high-resolution scans, respectively. The binding energy was calibrated to the TiO_2_ peak (458.5 eV) associated with the passive layer on the Si/Ti wafer support.^28^ Fits were carried out using commercial software (CasaXPS) after Shirley background subtraction and using mixed Gaussian–Lorentzian (30%) line shapes. Area uncertainties were estimated using Monte Carlo error analysis on Poisson adjusted spectra.

X-ray absorption near-edge structure (XANES) and extended X-ray absorption fine structure (EXAFS) measurements were undertaken at the SAMBA beamline at SOLEIL synchrotron, France.^29^ Reference samples of MnO, Mn_3_O_4_, α-Mn_2_O_3_, β-MnO_2_, and RuO_2_ (Sigma) were prepared as pellets using graphite powder and 5 wt % of the relevant Mn or Ru compound. Reference samples were probed in transmittance mode, while working electrodes prepared via thermal decomposition were probed in fluorescence mode at 45° unless otherwise noted. Spectra were collected at the Mn and Ru K-edges, and energy calibration was carried out using Mn (6540.0 eV) and Ru (22117.0 eV) metal foils as references.

Ex situ XAS spectra of oxide electrocatalysts were initially obtained from the as-prepared samples at the Mn K-edge. Ex situ electrochemical experiments were carried out in a three-electrode cell using 1 M NaOH as the electrolyte, a Pt wire as the counter electrode, and Hg/HgO as the reference electrode. Cyclic voltammetry was carried out at a scan rate of 40 mV/s. Chronopotentiometry was conducted at a current density of 10 mA cm^–2^. The samples were subsequently characterized again ex situ in the Mn K-edge. For the EXAFS analysis in the Mn K-edge region, data were processed to obtain the oscillatory χ(*k*) function by removing the background above the edge and was fit using the standard procedure. Briefly, the energy in electron volts (eV) was converted to k-space over the region from 3 to 10.5 Ǻ with a Hanning apodization window with sills of amplitude d*k* = 1. The data were then *k*^1^-weighted and Fourier-transformed to produce a pseudo-radial distribution function around Mn.

In situ and operando experiments at the Ru K-edge were carried out using a custom-built two-electrode cell (Figure S1) equipped with the Pt counter electrode that allowed for probing of the oxide through the Si/Ti substrate during OER activity in 1 M NaOH. Data analysis was performed using Athena and Artemis software packages. The edge position was determined from XANES as the energy at half the normalized edge absorbance. Calculation of scattering paths was carried out using FEFF v.8.4 and self-consistent potentials.^31^ EXAFS data were extracted as described by Newville et al.^29,30^ Fourier transforms (FT) were performed between *k* = 3 and 11 Å^–1^ with a Hanning apodization window with sills of amplitude d*k* = 1. The EXAFS signal was weighted by *k*^1^ and fitted in *r*-space according to the procedure described by Newville.^30^

## 3 Results and Discussion

In order to investigate the structural properties of the pure and mixed Mn/Ru oxide materials under OER conditions, the materials in this study were prepared by thermally annealing precursor salt materials at 350 °C in a tubular furnace. The materials were prepared similarly to our previous paper; however, due to the nature of the in situ/operando investigations in this current study, a flat Si/Ti support was used instead of a wire encapsulated in glass.^15^

**Figure 1:**
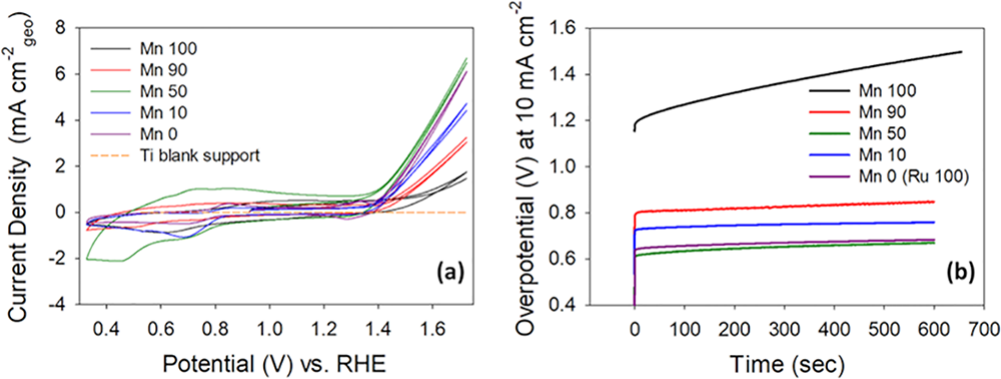
Electrochemical overview for the Mn 100, Mn 90, Mn 50, Mn 10, and Mn 0 materials on Si/Ti wafers in 1 M NaOH. (a) Cyclic voltammograms at a scan rate of 40 mV s^–1^ and (b) chronopotentiometry at a current density of 10 mA cm^–2^.

Typical cyclic voltammogram (CV) curves for the pure and mixed Mn/Ru oxide materials on the Si/Ti supports in 1 M NaOH can be observed in Figure 1a. From the CV curves, it is evident that the Mn 100 material is the least active material for the OER, while the Mn 50 material exhibits the highest OER current densities amongst all materials tested (Mn 100, Mn 90, Mn 50, Mn 10, and Mn 0) in this study. Furthermore, a plot of the CV curves after normalization by the respective capacitive contributions (see Figure S2) shows comparable or higher activity for Mn 50 and Mn 10 materials relative to the thermally prepared RuO_2_. This indicates that the improved OER current densities of mixed oxides are not likely to be due to differences in electrochemical specific surface area alone but rather to changes in intrinsic activity.

Chronopotentiometry measurements in the OER region (at a current density of 10 mA cm^–2^), shown in Figure 1b, were also conducted to evaluate the activity of the OER for the pure and mixed Mn/Ru oxides under more steady-state conditions, i.e., less influence of the capacitance current compared to the CV curves; all chronopotentiometry is shown as measured, in the absence of ohmic drop correction. For the chronopotentiometry measurements, the trend across Mn/Ru materials was consistent with OER activity from CV curves. Plots obtained after applying ohmic drop correction are also shown in the Supporting Information and indicate that the OER overpotentials of the mixed oxides are all either better than or comparable to that of the thermally prepared RuO_2_,^15^ as shown in Figure S3. Of further note is that all the mixed Mn/Ru materials are significantly better OER catalysts compared to the Mn 100 material.

The OER results in Figure 1 are extremely interesting as a state-of-the-art material (RuO_2_) which is diluted with 50% of an inexpensive material (Mn oxide) displays better OER activity than the RuO_2_ itself. Hence, catalyzing the OER with the Mn 50 rather than the RuO_2_ drives down the cost associated with this reaction. Furthermore, the structural design of the mixed Mn/Ru oxide materials is of particular interest as the information gained could be used to synthesize better and less expensive OER catalysts when compared to the state-of-the-art.

**Figure 2:**
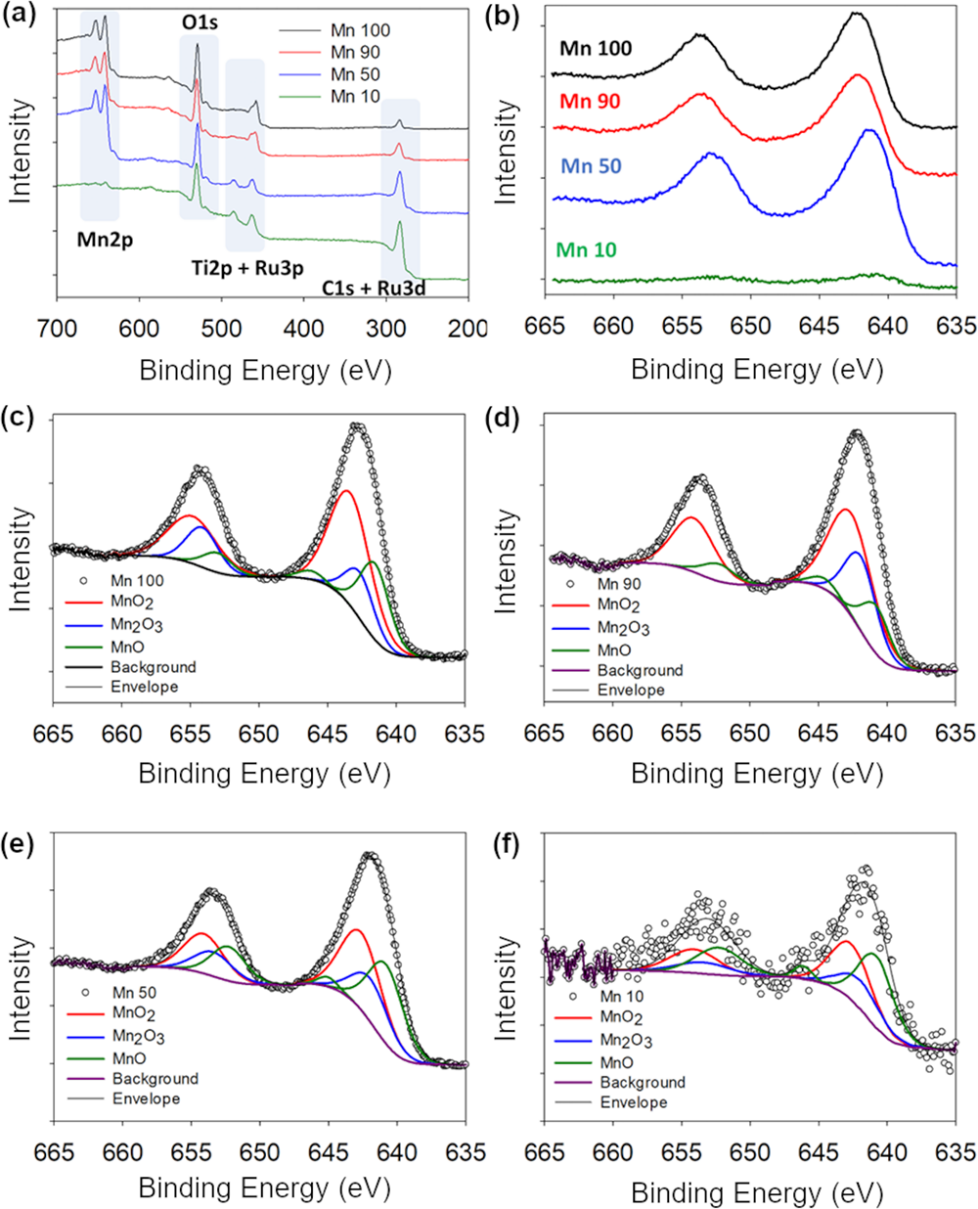
XPS analysis. (a) Survey spectra of the Mn 100–10 materials. (b) High-resolution Mn 2p core level for the Mn 100–10 materials. High-resolution Mn 2p spectra and best fits of Mn/Ru mixed oxides on Si/Ti wafers of (c) Mn 100, (d) Mn 90, (e) Mn 50, and (f) Mn 10.

To understand the structure and oxidation state of the metal centers at the surface and in the bulk of the pure and mixed Mn/Ru oxides, XPS and XAS measurements were carried out. Figure 2a shows survey scans of the materials synthesized with increasing proportions of Mn precursors; the survey of the material synthesized in the absence of Mn (Mn 0) is shown in Supporting Information Figure S4. All surveys show peaks associated with Ti 2p (ca. 458 eV)^28,31^ arising from the electrode substrate. C 1s peaks at ca. 285 eV result from residual carbon, while O 1s at ca. 532 eV arises from oxide formation. Mn-containing materials show Mn 2p doublets in the range of 635–660 eV,^32−35^ while Mn 0 and mixed Mn/Ru oxides exhibit Ru 3d (ca. 282 eV) and Ru 3p (ca. 464 eV) peaks^36^ that partially overlap with C 1s and Ti 2p contributions, respectively.

High-resolution spectra of the Mn 2p doublet of Mn/Ru oxides are shown in Figure 2b. Spectra display the characteristic broad doublet peaks of mixed valence manganese oxides.^32,34^ The binding energy of 2p_3/2_ maxima remains in the range of 640.7–642.2 eV for all oxides. This value suggests that the main contributions to the Mn 2p spectra arise from Mn centers with an oxidation state in the range of II–IV. Fittings of the Mn 2p doublets were carried out and are shown in Figure 2c–f; the results are summarized in Table 1. Best fits were obtained using three main contributions associated with Mn(IV) in MnO_2_ (642.0–642.6 eV), Mn(III) in Mn_2_O_3_ (641.3–641.9 eV), and Mn(II) in MnO (640.2–640.8 eV).^32^ Fits of the 2p_1/2_ peak shown in the figures were constrained in order to satisfy the 2:4 area ratio relative to the 2p_3/2_ and an energy split of 11.5 eV.^33,35^ A fourth peak at 644.5–646 eV, corresponding to the satellite peak of Mn(II), was also required to satisfactorily fit all spectra,^32,33^ further supporting the presence of Mn(II) centers in the materials. Fit results indicate that all materials display a mixed oxidation state; however, unambiguous determination of an average oxidation state from Mn 2p fits is challenging due to the peaks being broad and the energy shifts being relatively small.^32^

**Table 1:** Summary of XPS Results Obtained from Fits of the Mn 2p_3/2_ Spectrum^a^

	MnO_2_		Mn_2_O_3_		MnO		satellite	
	eV	% area	eV	% area	eV	% area	eV	% area
Mn 100	642.6	55 (1)%	641.9	16 (2)%	640.8	26 (2)%	645.5	2.6 (1.0)%
Mn 90	642.6	53 (1)%	641.9	28 (4)%	640.8	16 (3)%	644.6	3 (2)%
Mn 50	642.0	41 (1)%	641.3	21 (1)%	640.2	36 (1)%	644.5	2.4 (0.4)%
Mn 10	642.0	37 (10)%	641.3	19 (9)%	640.2	40 (8)%	645.6	4 (3)%

a Fit uncertainties are shown in parentheses.

### 3.1 XAS Characterization at the Mn K-Edge

**Figure 3:**
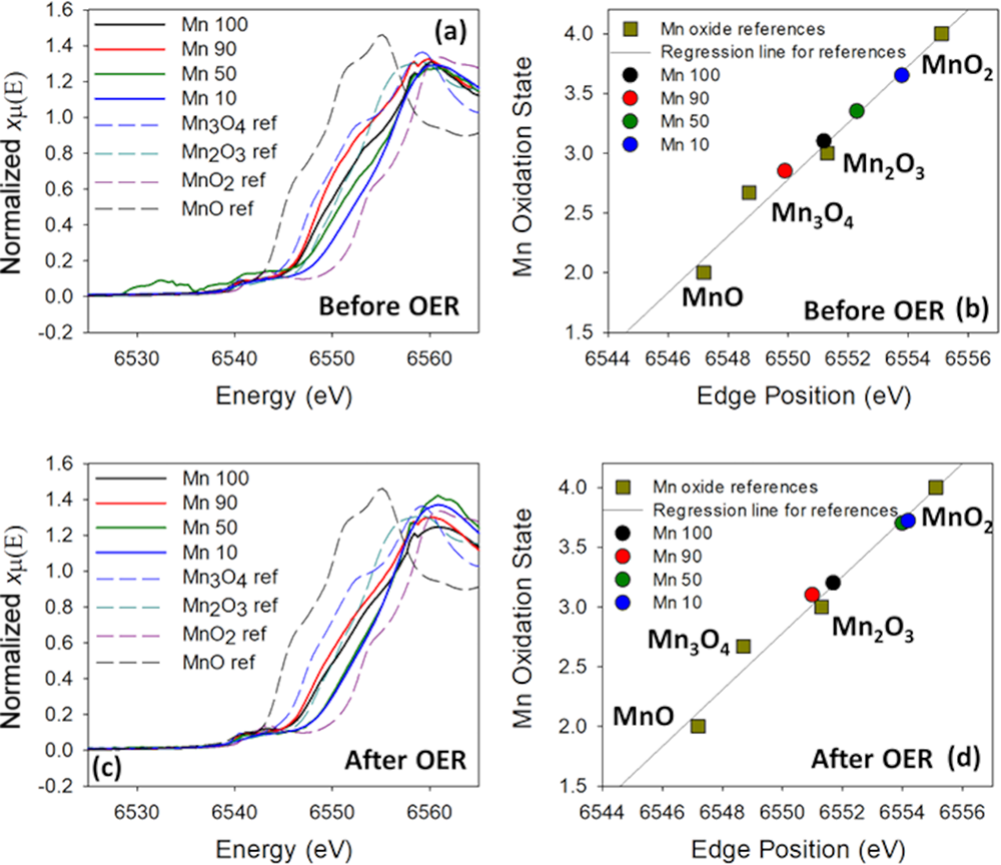
(a) XANES spectra of the as-prepared samples and reference Mn oxide materials. (b) Estimated Mn oxidation state based on the absorption edge position and interpolation of values for references MnO, Mn_3_O_4_, Mn_2_O_3_, and MnO_2_ before OER analysis. (c) XANES spectra of all electrocatalysts after OER tests and reference Mn oxide pristine materials. (d) Estimated Mn oxidation state based on the absorption edge position and interpolation of values for references MnO, Mn_3_O_4_, Mn_2_O_3_, and MnO_2_ after OER analysis.

XAS was used to investigate the local structure around Mn centers before and after OER activity. Normalized XANES spectra at the Mn K-edge of the as-prepared Mn/Ru mixed oxide materials are shown in Figure 3a; the XANES spectra of reference oxides MnO, Mn_3_O_4_, Mn_2_O_3_, and MnO_2_ are also shown for comparison; the oxidation states vs XANES edge position of the Mn oxide references can be seen in Table S1.

Figure 3b shows the estimated oxidation states obtained from a linear interpolation of the known oxidation states of the reference oxides.^21,37^ The edge position of the mixed Mn/Ru oxides suggests that the Mn oxidation state decreases with increasing Mn-content in the mixed oxide catalyst. Visible shoulders at ca. 6552 and 6555 eV are initially present in the mixed catalysts; such shoulders are normally observed for Mn oxides with a low oxidation state such as MnO,^20,37,38^ in agreement with the presence of a satellite in the Mn 2p spectra in Figure 2.

Initial oxidation states of mixed Mn/Ru oxides range from 2.8 to 3.6 following the sequence Mn 90, Mn 100, Mn 50, and Mn 10, as indicated in Figure 2b. The observed Mn valence in the pure Mn catalyst (Mn 100) closely matches the Mn valence of 3.0 observed in Mn_2_O_3_ and Mn_3_O_4_, indicating a higher contribution of Mn^3+^ than Mn^2+^. The XANES spectrum of Mn 90 exhibits a strong similarity to that of the Mn_3_O_4_ reference. The most active OER catalysts (Mn 10 and Mn 50) show edge positions suggestive of oxidation states >3.0 but <4.0, and their XANES spectra show similarities to that of MnO_2_, indicating the possible formation of birnessite, which has 20–40% Mn^3+^ centers in MnO_2_, with an average oxidation state of 3.6–3.8.^19^ It has been explicitly shown in the literature that an oxidation state above 3.0 but lower than 4.0 is optimal for catalysis of the OER with manganese-based materials.^39^ Therefore, the observed Mn oxidation states for the best performing mixed catalysts are consistent with previous findings.^39,40^

A significant upshift in the edge position was observed after the OER under galvanostatic conditions (Figure 3c), accompanied by a general suppression of shoulder contributions at ca. 6552 and 6555 eV. This suggests a significant change in the oxide local structure after the OER and a decrease in the proportion of Mn centers with a low oxidation state. A particularly significant change in the average oxidation state is observed for Mn 50, which appears nearly indistinguishable from Mn 10, as shown in Figure 3d and Table 2. It is important to note that the best OER material, Mn 50, exhibits an oxidation state of 3.7 after the OER, the optimum previously reported range of the best performing Mn oxides.^39^

**Table 2:** Mn-Edge Position and Estimated Oxidation State before and after OER Experiments

material	before OER		after OER		Δ oxidation state (after–before)
	edge position (eV)	Mn oxidation state	edge position (eV)	Mn oxidation state	
Mn 100	6551.2	3.1	6551.7	3.2	0.1
Mn 90	6549.9	2.8	6551.0	3.1	0.3
Mn 50	6552.3	3.4	6554.0	3.7	0.3
Mn 10	6553.8	3.6	6554.2	3.7	0.1

**Figure 4:**
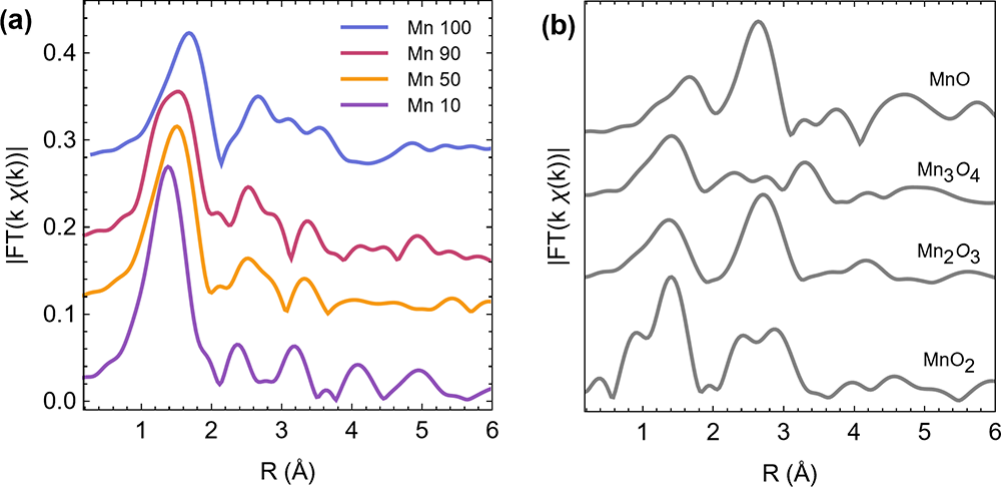
EXAFS collected at the Mn K-edge on (a) as-prepared catalysts Mn/Ru oxides studied and (b) references MnO, Mn_3_O_4_, Mn_2_O_3_, and MnO_2_. Fourier transforms shown are not phase corrected; curves are stacked to facilitate comparison.

The EXAFS spectra of Mn 10–100 samples (Figure S7) reveal further insights on the structure of these materials and the changes in the local structure after galvanostatic experiments in the OER potential region. Figure 4a shows the Fourier transform filtered *k*^1^-weighted EXAFS spectra (|*FT*(*k*^1^ χ(*k*)|) in real space of Mn/Ru mixed oxides prepared on Ti-coated Si wafers, while Figure 4b shows |*FT*(*k*^1^ χ(*k*)| in real space of reference Mn oxide compounds. Mn 100 exhibits peaks at various radial distances that could correspond to shells of MnO_2_ and Mn_3_O_4_. Significant scattering maxima at an upper distance of 1.3 Å are consistent with main peaks in MnO_2_; however, features at 2.3 and 3 Å can be found in either MnO_2_ or Mn_3_O_4_ references and are attributed to the first Mn–O–Mn coordination shell.^41,42^ The best fit of the first coordination shell (Figure S8, Table S2) yielded Mn–O distances of 1.89 Å, which are diagnostic for the presence of Mn^4+^ centers.^43^ However, a low apparent coordination number, well below 6, was also observed for the first shell indicating multiplicity in the oxidation state, site occupancy (layer, interlayer, edges), and/or type of ligand (−O, −OH, H_2_O), as previously reported for nanocrystalline phyllomanganates.^42,44−46^ Fit results are therefore consistent with Mn 100 being disordered and possessing an average oxidation state of 3.2, as a high proportion of Mn^3+^ or possibly Mn^2+^ sites in this material would be expected to shift Mn–O paths to *R*-values of 2 Å or larger.^42,43,46^

The spectra of Mn/Ru mixed oxides show broad peaks at ca. 1.3 Å associated with the first Mn–O coordination sphere.^41^ The peak at 2.3 Å present in all mixed oxides could be attributed to either MnO_2_ or Mn_3_O_4_; however, the feature at 3.1 Å, which is prominent in Mn 50 and Mn 10, clearly indicates the presence of Mn_3_O_4_-type oxides. Finally, the most prominent peak in the MnO reference spectrum at 2.7 Å, attributed to the Mn–O–Mn coordination shell,^41^ appears to be absent from all mixed oxide spectra. Best fits of the first coordination shell (Figure S8, Table S2) indicate that the peak at 1.3 Å results from relatively short Mn–O distances (1.85–1.89 Å) characteristic of Mn^4+^ centers. It is interesting to note that in the case of Mn 90, the coordination number is low and close to 3 as for Mn 100, in accordance with XANES results yielding a low oxidation state and consequently a high proportion of longer Mn–O distances. In contrast, Mn 10 and Mn 50 have coordination numbers closer to 4 at 1.89 Å, in agreement with their higher estimated oxidation states.^43^ In summary, Mn oxide electrocatalysts display disordered structures that are consistent with the presence of mixed oxidation states; the most active Mn 50 and Mn 10 materials display a disordered local structure and mixed valences with a greater contribution from Mn^4+^ centers compared to pure Mn 100 materials.

**Figure 5:**
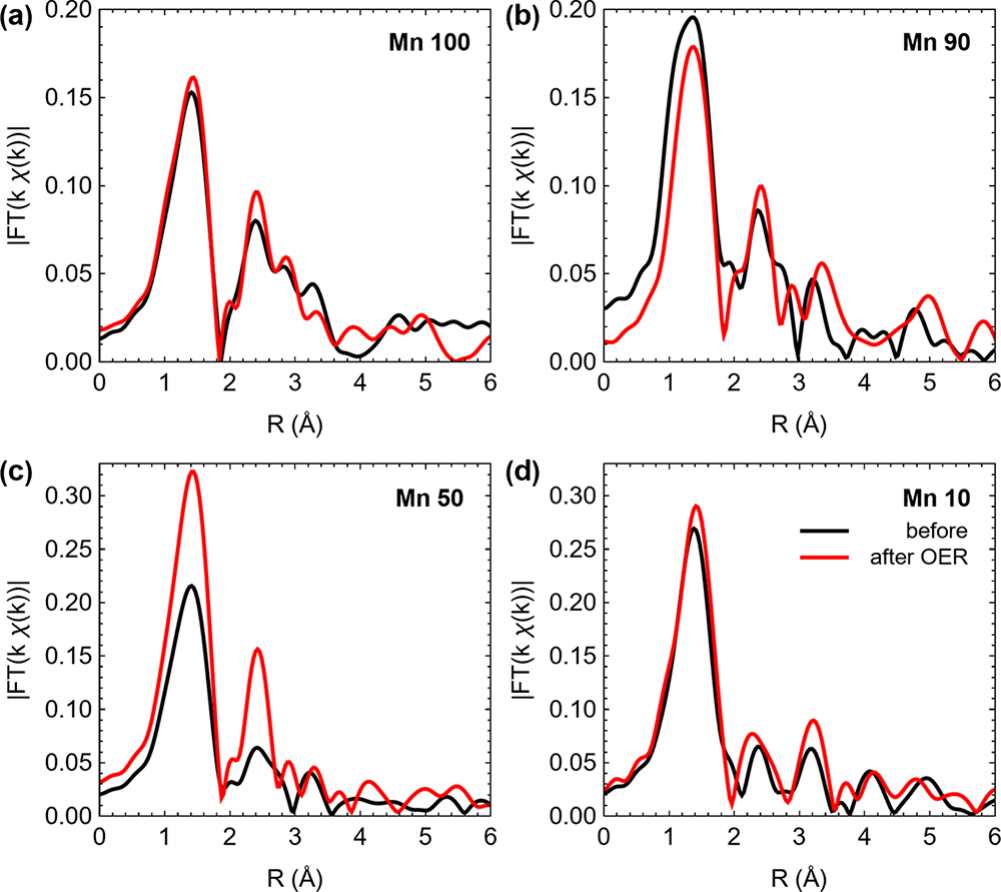
EXAFS Mn edge of the Mn 100–Mn 10 materials before and after the OER at 10 mA cm^–2^ on Si/Ti substrates: (a) Mn 100, (b) Mn 90, (c) Mn 50, and (d) Mn 10. Fourier transforms shown are not phase corrected.

The effect of OER activity on the Mn environment of the oxides was investigated using EXAFS analysis, and the results are shown in Figure 5; EXAFS spectra and best fits of the first coordination shell are reported in Table S3 and Figure S8. Figure 5a–d shows a comparison of the |*FT*(*k*^1^ χ(*k*)| in real space before and after OER galvanostatic experiments for Mn 100, Mn 90, Mn 50, and Mn 10 samples, respectively. The results indicate significant changes, particularly in the peaks corresponding to the first coordination shell for all mixed oxides. In the case of Mn 90, the first peak in the |*FT*(*k*^1^ χ(*k*)| shifts its mean position by ca. 0.2 Å. The |*FT*(*k*^1^ χ(*k*)| of Mn 50 shows a large increase in amplitude (Figure 5c) so that the coordination sphere after the OER qualitatively resembles that of the MnO_2_ reference material (Figure 4b). This is also suggested by the change in the coordination number for the shortest Mn–O distance (Tables S2 and S3), which increases to a value close to 6 while its *R*-value remains constant, thus suggesting that after the OER, Mn 50 has a higher proportion of Mn^4+^ centers. This is consistent with the increase in the estimated average oxidation state observed in Figure 3d and supports the conclusion that OER activity results in changes in the local structure of Mn in mixed oxide materials and in particular an increase in the average oxidation state.

### 3.2 XAS Characterization at the Ru K-Edge

**Figure 6:**
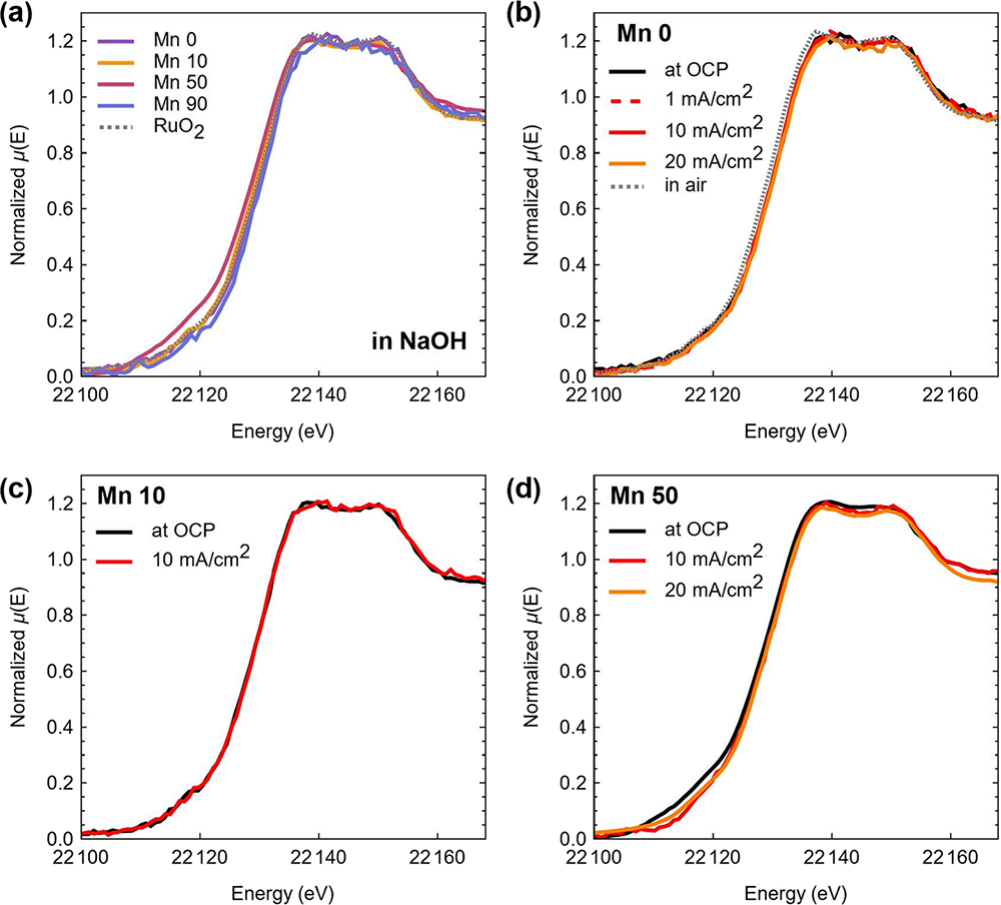
Ru K-edge. (a) Comparison of XANES for Mn 0–90 in NaOH and RuO_2_ reference. (b) Comparison of XANES for the Mn 0 ex situ in NaOH at OCP and at 1, 10, and 20 mA cm^–2^. (c) Comparison of XANES for the Mn 10 in NaOH at OCP and at 10 mA cm^–2^. (d) Comparison of XANES for the Mn 50 in NaOH and at 10 and 20 mA cm^–2^.

Further XAS measurements were performed at the Ru K-edge on the thermally prepared pure Ru oxide (Mn 0), mixed Mn/Ru oxide materials, and a RuO_2_ reference to gain insight into OER activity in these materials. Figure 6a shows the XANES spectra of Mn 0–90 obtained while immersed in the NaOH electrolyte solution at open circuit potential (OCP); the XANES of a commercially sourced RuO_2_ is also shown for comparison. The Ru edge position shows a spread of ca. 1.5 eV (Table S4) as expected from the relatively large width of the Ru core hole.^47^ Edge positions are at ca. 22129 eV: this is below the values of ca. 22,132 and 22,134 eV for Ru^5+^ and Ru^6+^, respectively, determined by Tarascon and co-workers,^48^ and close to the edge position measured for our RuO_2_ reference, thus suggesting likely oxidation states of +4 for all mixed oxides.

In order to gain an understanding of the oxidation state during the OER, operando XANES measurements of the Mn 0 catalyst were conducted by applying currents of 1, 10, and 20 mA cm^–2^ (Figure 6b). No shift in the edge position or changes in the spectral profile were evident with increasing current in the OER region. Similarly, no changes in the edge position were detected for the Mn 10 or Mn 50 during OER activity at 10 mA cm^–2^ current outputs, as shown in Figure 6c,d. These results do not suggest significant changes in the oxidation state for the majority of probed Ru centers in either Mn 0 or mixed oxides during the OER.

**Figure 7:**
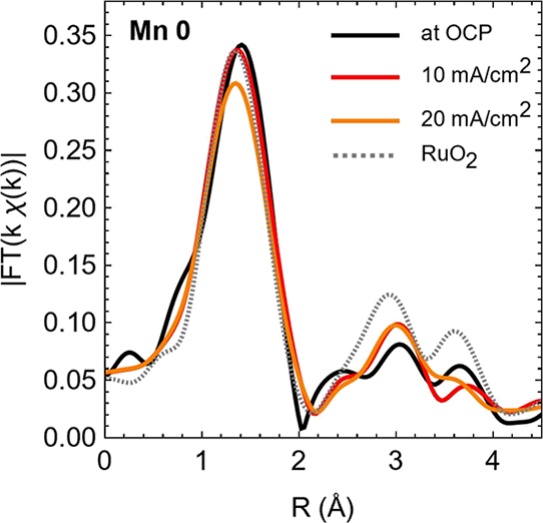
EXAFS at the Ru K-edge. Comparison of the Mn 0 (Ru 100) in NaOH at OCP and at OER currents of 10 and 20 mA cm^–2^; the RuO_2_ reference is also shown for comparison. Fourier transforms shown are not phase corrected.

In situ EXAFS analysis was undertaken at the Ru K-edge to further examine the pure and mixed Mn/Ru oxides for any changes in the local structure that could be linked to the OER performance (Figure S9). The structure of the Mn 0 (Ru 100) sample was investigated by comparing it to the reference RuO_2_ (Figure 7); the local structure of the Mn 0 catalyst appears to involve RuO_6_ coordination in the first shell, as is the case in rutile, but differs from that of crystalline RuO_2_ based on deviations in peaks at longer *R*-values (ca. 3.2 Å) associated with Ru–Ru distances. Octahedral coordination was supported also by a fit of the first shell (Figure S10, Table S5), which yielded an average Ru–O_1_ distance of 1.96 Å in good agreement with the literature.^49^ Upon applying OER currents of 10 and 20 mA cm^–2^, limited changes can be observed in the Fourier transform of the first coordination sphere; best fits suggest that Ru centers maintain average RuO_6_ coordination with ligands at ca. 1.96 Å during O_2_ evolution. The Ru–Ru contributions approach the position and height of the reference RuO_2_ material.

**Figure 8:**
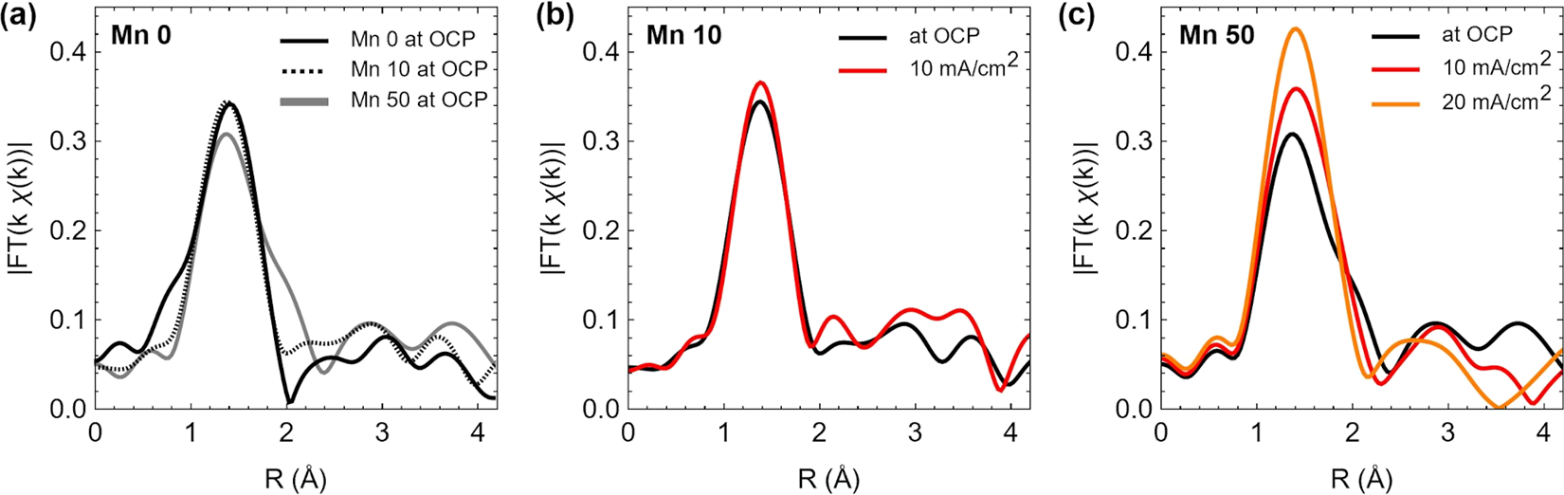
EXAFS at the Ru K-edge. (a) Comparison of spectra obtained at the Ru K-edge for the Mn 0–50 electrodes in NaOH solution at OCP. The mixed Mn/Ru electrodes in NaOH solution and at 10 and 20 mA cm^–2^, where applicable, for (b) Mn 10 and (c) Mn 50. Fourier transforms shown are not phase corrected.

The Mn 0 EXAFS Ru-edge curve was compared to that of the mixed Mn 10 and Mn 50 Mn/Ru oxides; Figure 8a shows this comparison for the samples immersed in 1.0 M NaOH. The structure of Mn 10 shows the main features of the RuO_2_ structure in Mn 0 samples, with the first coordination shell corresponding to Ru–O and contributions at 3.2–3.6 Å arising from the Ru–Ru distances. In the case of Mn 50, the main coordination spheres are still evident from the spectrum, but changes in the degree of order relative to the Mn 0 structure are readily apparent. Fits of the first coordination shell indicate that octahedral coordination is maintained for both Mn 10 and 50; however, the average Ru–O distances are slightly larger in these mixed oxides when compared to Mn 0 samples (Figure S10, Table S5), likely due to local disorder. Also, in the case of Mn 50, large deviations are particularly evident for peaks positioned at >2 Å. This is partly due to the presence of Ru metal^50^ that was detected in our Mn 50 sample by XRD in our previous work^15^ and that is consistent with the analysis from linear combination fits of the XANES spectrum (Figure S11 and Table S6). For Mn 90 samples, the EXAFS signal was weaker (data not shown) due to this being the sample with the lowest Ru %-content, which prevented a detailed analysis of its structure.

The EXAFS spectra of the mixed Mn/Ru catalysts were recorded during the OER at a current of 10 mA cm^–2^ (Figure 8) and also at 20 mA cm^–2^ for Mn 50 (Figure S9). In the case of Mn 10 (Figure 8b), the changes are comparable to those observed for Mn 0 samples, i.e., the radial distances of peaks at 1.9, 3.2, and 3.6 Å are not altered during the OER, while the changes in the intensity of the peaks suggest a limited reorganization of the first O-coordination and Ru–Ru shells. For highly active Mn 50 samples (Figure 8c), the EXAFS signal is significantly different under O_2_ evolution compared to the sample in NaOH and indicates significant structural rearrangement around Ru centers. During the OER at 10 mA cm^–2^, the Ru metal peak is extremely evident in the Fourier transform, and from linear combination fits using RuO_2_ and Ru metal foil references (Figure S11 and Table S5), it was calculated that Ru metal is still present in the Mn 50 sample during the OER. It is interesting to note that the largest changes in the local structure are observed for Mn 50, i.e., the sample with the largest Mn-content that could be successfully characterized in operando. This is likely due to the majority of the Ru-edge signal arising from highly active Ru centers, for which local rearrangements become more evident and less obscured by the presence of a rutile-like RuO_2_ phase (as in Mn 0). Notably, best fits of the first shell indicate an increase in the average Ru–O coordination number, which becomes more pronounced at increasing current density (Figure S10, Table S5). Despite the large uncertainties associated with the number of first-neighbors, the general trend suggests significant coordination flexibility in the highly active Mn/Ru 50:50 oxide and the formation of high-coordinated Ru species during oxygen evolution. This might possibly involve an increase in the oxidation state given the limited change in the first Ru–O distance.

Adaptive ligand spheres with *N* > 6 are recognized to be critical for the design of high-turnover homogeneous OER catalysts;^51^ it is therefore intriguing that our observations suggest an expansion of the O-coordination sphere under reaction conditions also in the case of these mixed oxide electrocatalysts. Ligand sphere expansion is likely accompanied by stabilization of oxidation states greater than +4, as previously highlighted in the organometallic literature;^51^ however, it is difficult to confirm whether this indeed occurs during the OER in Mn 50, given the limited changes observed in the Ru-edge XANES.

## 4 Conclusions

In agreement with previous studies in the literature, the CV and chronopotentiometry measurements in this study show that mixed Mn/Ru oxides show great potential to replace the more expensive RuO_2_ catalysts for the OER. In particular, the less expensive Mn 50 and Mn 10 show good OER activity that is competitive with that of the pure RuO_2_ catalyst.

In a bid to understand why these oxides can outperform pure RuO_2_ catalysts, ex situ and in situ XAS was employed to determine the oxidation state and coordination shells of both types of metal centers in the mixed oxides as a function of metal composition. XANES analysis of the Mn K-edge reveals that the average Mn oxidation state in the as-prepared materials decreases with increasing amounts of Mn in the mixed Mn/Ru oxides, i.e., the Mn 10 exhibits the highest oxidation state, while Ru K-edge positions suggest the presence of Ru centers with an oxidation state of +4. From the EXAFS measurements, the first Mn–O ligand sphere indicates the presence of Mn^4+^ centers in all mixed oxides but in a decreasing proportion with decreasing Ru-content. Importantly, a high degree of structural disorder was observed in Mn 50; the Mn local structure in this oxide in fact undergoes significant changes after the OER, which suggests an increase in the concentration of Mn^4+^ centers relative to the pristine samples.

A study of the Ru centers was also carried out under operando conditions to monitor changes in the local structure during oxygen evolution. The operando EXAFS analysis at the Ru edge indicates that Mn 50, in particular, undergoes changes in the local structure during the OER at high currents. Best fits are suggestive of perturbations in the first coordination shell through expansion of the ligand sphere. We hypothesize that this is likely to stabilize Ru at higher oxidation states; however, XANES spectra do not show significant changes that might be expected to accompany this.

Based on our results, it is interesting to speculate on the role of the MnO_*x*_ phase in imparting high OER activity in binary or mixed Mn/Ru oxides. Based on the EXAFS/XANES results, it appears that significant structural changes during the OER are taking place at Ru and Mn centers. The role of the MnO_*x*_ phase might be two-fold: first, we note that the mixing with RuO_*x*_ affects the average oxidation state of Mn centers, and it is therefore possible that this tuning effect is partly responsible for activity enhancements in, e.g., Mn 10 or Mn 50 materials. Second, the local structure around Ru centers in mixed oxides appears to be highly disordered and for the best performing mixed oxides undergoes changes during oxygen evolution, with the MnO_*x*_ content possibly imparting greater coordination flexibility around the active Ru sites.

This study contributes important insights on how to tailor the MnO_*x*_ content in mixed oxide electrocatalysts for the OER. In particular, the lower cost and the improved OER performance of the Mn 50 and Mn 10 materials compared to the pure RuO_2_ make these catalysts an attractive and cost-competitive choice for water splitting applications.

## Data Availability

The data related to the findings of this work are available from the corresponding authors, subject to reasonable request.

## Supporting Information Available

Additional chronoamperometry, cyclic voltammetry, X-ray photoelectron spectroscopy, X-ray diffraction, EXAFS spectra, best fits and parameters for the first coordination shell, and fit results of the XANES spectrum of Mn 50 (PDF)

## References

[ref1] Zeng K.; Zhang D. Recent progress in alkaline water electrolysis for hydrogen production and applications. Prog. Energy Combust. Sci. 2010, 36, 307–326. 10.1016/j.pecs.2009.11.002.

[ref2] Rao R. R.; Kolb M. J.; Halck N. B.; Pedersen A. F.; Mehta A.; You H.; Stoerzinger K. A.; Feng Z.; Hansen H. A.; Zhou H.; Giordano L.; Rossmeisl J.; Vegge T.; Chorkendorff I.; Stephens I. E. L.; Shao-Horn Y. Towards identifying the active sites on RuO_2_(110) in catalyzing oxygen evolution. Energy Environ. Sci. 2017, 10, 2626–2637. 10.1039/C7EE02307C.

[ref3] Demirbas A. Future hydrogen economy and policy. Energy Sources, Part B 2017, 12, 172–181. 10.1080/15567249.2014.950394.

[ref4] Benck J. D.; Hellstern T. R.; Kibsgaard J.; Chakthranont P.; Jaramillo T. F. Catalyzing the Hydrogen Evolution Reaction (HER) with Molybdenum Sulfide Nanomaterials. ACS Catal. 2014, 4, 3957–3971. 10.1021/cs500923c.

[ref5] Shi X.; Fields M.; Park J.; McEnaney J. M.; Yan H.; Zhang Y.; Tsai C.; Jaramillo T. F.; Sinclair R.; Nørskov J. K.; Zheng X. Rapid flame doping of Co to WS_2_ for efficient hydrogen evolution. Energy Environ. Sci. 2018, 11, 2270–2277. 10.1039/C8EE01111G.

[ref6] Lyons M. E. G.; Doyle R. L.; Browne M. P.; Godwin I. J.; Rovetta A. A. S. Recent developments in electrochemical water oxidation. Curr. Opin. Electrochem. 2017, 1, 40–45. 10.1016/j.coelec.2016.12.005.

[ref7] Cherevko S.; Geiger S.; Kasian O.; Kulyk N.; Grote J.-P.; Savan A.; Shrestha B. R.; Merzlikin S.; Breitbach B.; Ludwig A.; Mayrhofer K. J. J. Oxygen and hydrogen evolution reactions on Ru, RuO_2_, Ir, and IrO_2_ thin film electrodes in acidic and alkaline electrolytes: A comparative study on activity and stability. Catal. Today 2016, 262, 170–180. 10.1016/j.cattod.2015.08.014.

[ref8] Sayeed M. A.; Herd T.; O’Mullane A. P. Direct electrochemical formation of nanostructured amorphous Co(OH)_2_ on gold electrodes with enhanced activity for the oxygen evolution reaction. J. Mater. Chem. A 2016, 4, 991–999. 10.1039/C5TA09125J.

[ref9] Pei Y.; Yang Y.; Zhang F.; Dong P.; Baines R.; Ge Y.; Chu H.; Ajayan P. M.; Shen J.; Ye M. Controlled Electrodeposition Synthesis of Co–Ni–P Film as a Flexible and Inexpensive Electrode for Efficient Overall Water Splitting. ACS Appl. Mater. Interfaces 2017, 9, 31887–31896. 10.1021/acsami.7b09282. 28849904

[ref10] Escudero-Escribano M.; Pedersen A. F.; Paoli E. A.; Frydendal R.; Friebel D.; Malacrida P.; Rossmeisl J.; Stephens I. E. L.; Chorkendorff I. Importance of Surface IrO_*x*_ in Stabilizing RuO_2_ for Oxygen Evolution. J. Phys. Chem. B 2018, 122, 947–955. 10.1021/acs.jpcb.7b07047. 29045788

[ref11] Browne M. P.; O’Rourke C.; Mills A. A mechanical, high surface area and solvent-free ‘powder-to-electrode’ fabrication method for screening OER catalysts. Electrochem. Commun. 2017, 85, 1–5. 10.1016/j.elecom.2017.10.011.

[ref12] Stoerzinger K. A.; Qiao L.; Biegalski M. D.; Shao-Horn Y. Orientation-Dependent Oxygen Evolution Activities of Rutile IrO_2_ and RuO_2_. J. Phys. Chem. Lett. 2014, 5, 1636–1641. 10.1021/jz500610u. 26270358

[ref13] Gao X.; Zhang H.; Li Q.; Yu X.; Hong Z.; Zhang X.; Liang C.; Lin Z. Hierarchical NiCo_2_O_4_ Hollow Microcuboids as Bifunctional Electrocatalysts for Overall Water-Splitting. Angew. Chem., Int. Ed. 2016, 55, 6290–6294. 10.1002/anie.201600525. 27061909

[ref14] Etzi Coller Pascuzzi M.; Goryachev A.; Hofmann J. P.; Hensen E. J. M. Mn promotion of rutile TiO_2_-RuO_2_ anodes for water oxidation in acidic media. Appl. Catal., B 2020, 261, 118225 10.1016/j.apcatb.2019.118225.

[ref15] Browne M. P.; Nolan H.; Duesberg G. S.; Colavita P. E.; Lyons M. E. G. Low-Overpotential High-Activity Mixed Manganese and Ruthenium Oxide Electrocatalysts for Oxygen Evolution Reaction in Alkaline Media. ACS Catal. 2016, 6, 2408–2415. 10.1021/acscatal.5b02069.

[ref16] Browne M. P.; Nolan H.; Twamley B.; Duesberg G. S.; Colavita P. E.; Lyons M. E. G. Thermally Prepared Mn_2_O_3_/RuO_2_/Ru Thin Films as Highly Active Catalysts for the Oxygen Evolution Reaction in Alkaline Media. ChemElectroChem 2016, 3, 1847–1855. 10.1002/celc.201600370.

[ref17] Wu Y.; Tariq M.; Zaman W. Q.; Sun W.; Zhou Z.; Yang J. Bimetallic Doped RuO_2_ with Manganese and Iron as Electrocatalysts for Favorable Oxygen Evolution Reaction Performance. ACS Omega 2020, 5, 7342–7347. 10.1021/acsomega.9b04237. 32280875 PMC7144150

[ref18] Lin C.; Li J.-L.; Li X.; Yang S.; Luo W.; Zhang Y.; Kim S.-H.; Kim D.-H.; Shinde S. S.; Li Y.-F.; Liu Z.-P.; Jiang Z.; Lee J.-H. In-situ reconstructed Ru atom array on α-MnO_2_ with enhanced performance for acidic water oxidation. Nat. Catal. 2021, 4, 1012–1023. 10.1038/s41929-021-00703-0.

[ref19] Gorlin Y.; Lassalle-Kaiser B.; Benck J. D.; Gul S.; Webb S. M.; Yachandra V. K.; Yano J.; Jaramillo T. F. In situ X-ray absorption spectroscopy investigation of a bifunctional manganese oxide catalyst with high activity for electrochemical water oxidation and oxygen reduction. J. Am. Chem. Soc. 2013, 135, 8525–8534. 10.1021/ja3104632. 23758050 PMC3874100

[ref20] Frydendal R.; Seitz L. C.; Sokaras D.; Weng T.-C.; Nordlund D.; Chorkendorff I.; Stephens I. E. L.; Jaramillo T. F. Operando investigation of Au-MnO_*x*_ thin films with improved activity for the oxygen evolution reaction. Electrochim. Acta 2017, 230, 22–28. 10.1016/j.electacta.2017.01.085.

[ref21] Lian S.; Browne M. P.; Domínguez C.; Stamatin S. N.; Nolan H.; Duesberg G. S.; Lyons M. E. G.; Fonda E.; Colavita P. E. Template-free synthesis of mesoporous manganese oxides with catalytic activity in the oxygen evolution reaction. Sustainable Energy Fuels 2017, 1, 780–788. 10.1039/C7SE00086C.

[ref22] Petrykin V.; Bastl Z.; Franc J.; Macounova K.; Makarova M.; Mukerjee S.; Ramaswamy N.; Spirovova I.; Krtil P. Local Structure of Nanocrystalline Ru_1–*x*_Ni_*x*_O_2_–δ Dioxide and Its Implications for Electrocatalytic Behavior—An XPS and XAS Study. J. Phys. Chem. C 2009, 113, 21657–21666. 10.1021/jp904935e.

[ref23] Seitz L. C.; Nordlund D.; Gallo A.; Jaramillo T. F. Tuning Composition and Activity of Cobalt Titanium Oxide Catalysts for the Oxygen Evolution Reaction. Electrochim. Acta 2016, 193, 240–245. 10.1016/j.electacta.2016.01.200.

[ref24] Abbott D. F.; Lebedev D.; Waltar K.; Povia M.; Nachtegaal M.; Fabbri E.; Copéret C.; Schmidt T. J. Iridium Oxide for the Oxygen Evolution Reaction: Correlation between Particle Size, Morphology, and the Surface Hydroxo Layer from Operando XAS. Chem. Mater. 2016, 28, 6591–6604. 10.1021/acs.chemmater.6b02625.

[ref25] Gorlin Y.; Chung C.-J.; Benck J. D.; Nordlund D.; Seitz L.; Weng T.-C.; Sokaras D.; Clemens B. M.; Jaramillo T. F. Understanding Interactions between Manganese Oxide and Gold That Lead to Enhanced Activity for Electrocatalytic Water Oxidation. J. Am. Chem. Soc. 2014, 136, 4920–4926. 10.1021/ja407581w. 24661269 PMC4004245

[ref26] Petrykin V.; Macounová K.; Okube M.; Mukerjee S.; Krtil P. Local structure of Co doped RuO_2_ nanocrystalline electrocatalytic materials for chlorine and oxygen evolution. Catal. Today 2013, 202, 63–69. 10.1016/j.cattod.2012.03.075.

[ref27] Petrykin V.; Macounova K.; Shlyakhtin O. A.; Krtil P. Tailoring the Selectivity for Electrocatalytic Oxygen Evolution on Ruthenium Oxides by Zinc Substitution. Angew. Chem., Int. Ed. 2010, 49, 4813–4815. 10.1002/anie.200907128. 20514655

[ref28] Biesinger M. C.; Lau L. W. M.; Gerson A. R.; Smart R. S. C. Resolving surface chemical states in XPS analysis of first row transition metals, oxides and hydroxides: Sc, Ti, V, Cu and Zn. Appl. Surf. Sci. 2010, 257, 887–898. 10.1016/j.apsusc.2010.07.086.

[ref29] Newville M. EXAFS analysis using FEFF and FEFFIT. J. Synchrotron Radiat. 2001, 8, 96–100. 10.1107/S0909049500016290. 11512993

[ref30] Newville M. IFEFFIT: interactive XAFS analysis and FEFF fitting. J. Synchrotron Radiat. 2001, 8, 322–324. 10.1107/S0909049500016964. 11512767

[ref31] Diebold U.; Madey T. E. TiO_2_ by XPS. Surf. Sci. Spectra 1996, 4, 227–231. 10.1116/1.1247794.

[ref32] Di Castro V.; Polzonetti G. XPS study of MnO oxidation. J. Electron Spectrosc. Relat. Phenom. 1989, 48, 117–123. 10.1016/0368-2048(89)80009-X.

[ref33] Biesinger M. C.; Payne B. P.; Grosvenor A. P.; Lau L. W. M.; Gerson A. R.; Smart R. S. C. Resolving surface chemical states in XPS analysis of first row transition metals, oxides and hydroxides: Cr, Mn, Fe, Co and Ni. Appl. Surf. Sci. 2011, 257, 2717–2730. 10.1016/j.apsusc.2010.10.051.

[ref34] Wöllner A.; Lange F.; Schmelz H.; Knözinger H. Characterization of mixed copper-manganese oxides supported on titania catalysts for selective oxidation of ammonia. Appl. Catal., A 1993, 94, 181–203. 10.1016/0926-860X(93)85007-C.

[ref35] Wei Y. J.; Yan L. Y.; Wang C. Z.; Xu X. G.; Wu F.; Chen G. Effects of Ni Doping on [MnO_6_] Octahedron in LiMn_2_O_4_. J. Phys. Chem. B 2004, 108, 18547–18551. 10.1021/jp0479522.

[ref36] Morgan D. J. Resolving ruthenium: XPS studies of common ruthenium materials. Surf. Interface Anal. 2015, 47, 1072–1079. 10.1002/sia.5852.

[ref37] Jiao F.; Frei H. Nanostructured manganese oxide clusters supported on mesoporous silica as efficient oxygen-evolving catalysts. Chem. Commun. 2010, 46, 2920–2922. 10.1039/B921820C. 20386823

[ref38] Farges F. Ab initio and experimental pre-edge investigations of the Mn K-edge XANES in oxide-type materials. Phys. Rev. B 2005, 71, 155109 10.1103/PhysRevB.71.155109.

[ref39] Ramírez A.; Hillebrand P.; Stellmach D.; May M. M.; Bogdanoff P.; Fiechter S. Evaluation of MnO_*x*_, Mn_2_O_3_, and Mn_3_O_4_ Electrodeposited Films for the Oxygen Evolution Reaction of Water. J. Phys. Chem. C 2014, 118, 14073–14081. 10.1021/jp500939d.

[ref40] Mattelaer F.; Bosserez T.; Rongé J.; Martens J. A.; Dendooven J.; Detavernier C. Manganese oxide films with controlled oxidation state for water splitting devices through a combination of atomic layer deposition and post-deposition annealing. RSC Adv. 2016, 6, 98337–98343. 10.1039/C6RA19188F.

[ref41] Liu F.; Shan W.; Lian Z.; Xie L.; Yang W.; He H. Novel MnWO_*x*_ catalyst with remarkable performance for low temperature NH_3_-SCR of NO_*x*_. Catal. Sci. Technol. 2013, 3, 2699–2707. 10.1039/C3CY00326D.

[ref42] Wiechen M.; Zaharieva I.; Dau H.; Kurz P. Layered manganese oxides for water-oxidation: alkaline earth cations influence catalytic activity in a photosystem II-like fashion. Chem. Sci. 2012, 3, 2330–2339. 10.1039/C2SC20226C.

[ref43] Zahoransky T.; Wegorzewski A. V.; Huong W.; Mikutta C. X-ray absorption spectroscopy study of Mn reference compounds for Mn speciation in terrestrial surface environments. Am. Mineral. 2023, 108, 847–864. 10.2138/am-2022-8236.

[ref44] Grangeon S.; Lanson B.; Miyata N.; Tani Y.; Manceau A. Structure of nanocrystalline phyllomanganates produced by freshwater fungi. Am. Mineral. 2010, 95, 1608–1616. 10.2138/am.2010.3516.

[ref45] Zhang A.; Zhao R.; Hu L.; Yang R.; Yao S.; Wang S.; Yang Z.; Yan Y.-M. Adjusting the Coordination Environment of Mn Enhances Supercapacitor Performance of MnO_2_. Adv. Energy Mater. 2021, 11, 2101412 10.1002/aenm.202101412.

[ref46] Gaillot A.-C.; Flot D.; Drits V. A.; Manceau A.; Burghammer M.; Lanson B. Structure of Synthetic K-rich Birnessite Obtained by High-Temperature Decomposition of KMnO_4_. I. Two-Layer Polytype from 800 °C Experiment. Chem. Mater. 2003, 15, 4666–4678. 10.1021/cm021733g.

[ref47] Krause M. O.; Oliver J. H. Natural widths of atomic K and L levels, K_α_ X-ray lines and several KLL Auger lines. J. Phys. Chem. Ref. Data 1979, 8, 329–338. 10.1063/1.555595.

[ref48] Otoyama M.; Jacquet Q.; Iadecola A.; Saubanère M.; Rousse G.; Tarascon J.-M. Synthesis and Electrochemical Activity of Some Na(Li)-Rich Ruthenium Oxides with the Feasibility to Stabilize Ru^6+^. Adv. Energy Mater. 2019, 9, 1803674 10.1002/aenm.201803674.

[ref49] McKeown D. A.; Hagans P. L.; Carette L. P. L.; Russell A. E.; Swider K. E.; Rolison D. R. Structure of Hydrous Ruthenium Oxides: Implications for Charge Storage. J. Phys. Chem. B 1999, 103, 4825–4832. 10.1021/jp990096n.

[ref50] Qiu J.-Z.; Hu J.; Lan J.; Wang L.-F.; Fu G.; Xiao R.; Ge B.; Jiang J. Pure Siliceous Zeolite-Supported Ru Single-Atom Active Sites for Ammonia Synthesis. Chem. Mater. 2019, 31, 9413–9421. 10.1021/acs.chemmater.9b03099.

[ref51] Matheu R.; Ertem M. Z.; Gimbert-Suriñach C.; Sala X.; Llobet A. Seven Coordinated Molecular Ruthenium–Water Oxidation Catalysts: A Coordination Chemistry Journey. Chem. Rev. 2019, 119, 3453–3471. 10.1021/acs.chemrev.8b00537. 30816700

