# Supplementary material for: Probing Changes in the Local Structure of Active Bimetallic Mn/Ru Oxides during Oxygen Evolution
Source: ACS Appl Energy Mater. 2023 Aug 16;6(16):8607–15. doi: 10.1021/acsaem.3c01585 (PMC10466265; doi:10.1021/acsaem.3c01585)
Supplement: Supplementary file 1 — ae3c01585_si_001.pdf [file ae3c01585_si_001.pdf]

Supporting Information

Probing changes in local structure of active bimetallic Mn/Ru oxides during oxygen evolution

Michelle P. Browne,[ab*] *Carlota Domínguez,*[a] *Can Kaplan,*[b] *Michael E. G. Lyons,*[a] *Emiliano Fonda*[c] *and Paula E. Colavita*[a*]

[a] School of Chemistry, CRANN and AMBER Research Centres, Trinity College Dublin, College Green, Dublin D02 PN40, Ireland.

[b] Helmholtz Young Investigator Group Electrocatalysis: Synthesis to Devices, Helmholtz-Zentrum Berlin für Materialien und Energie, 14109 Berlin, Germany.

[c] SAMBA Beamline, SOLEIL Synchrotron, L'Orme des Merisiers, Saint-Aubin, BP48, 91192 Gif-sur-Yvette, France.

Email corresponding authors: colavitp@tcd.ie; michelle.browne@helmholtz-berlin.de

Keywords: X-Ray Absorption Spectroscopy, Oxygen Evolution Reaction, operando, mixed oxides, water splitting

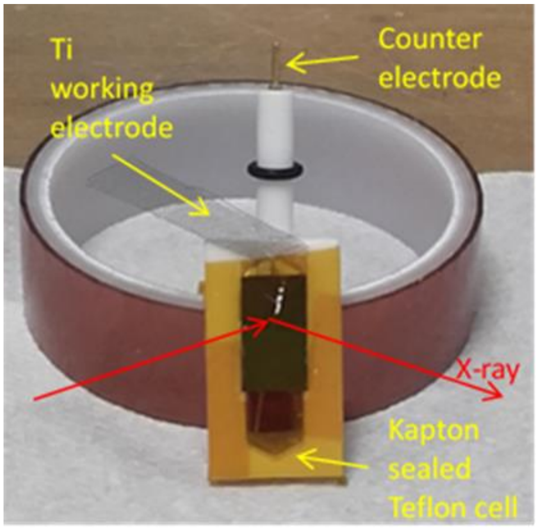

Figure S1. Schematic of the in-house XAS electrochem cell

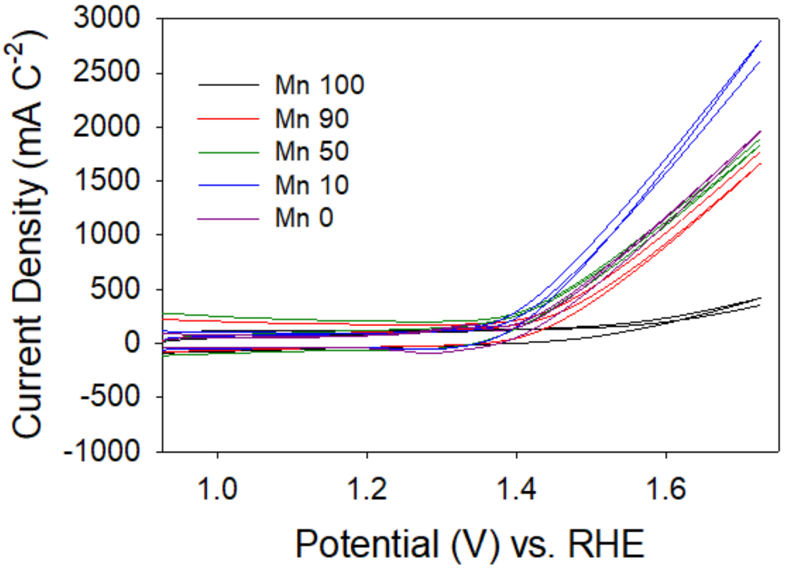

Figure S2. CVs normalised by capacitive contributions obtained from charge integration over the potential window outside the faradaic region.[1] The figure shows that mixed Mn/Ru oxides display similar or better performance than the RuO_2 (Mn 0).

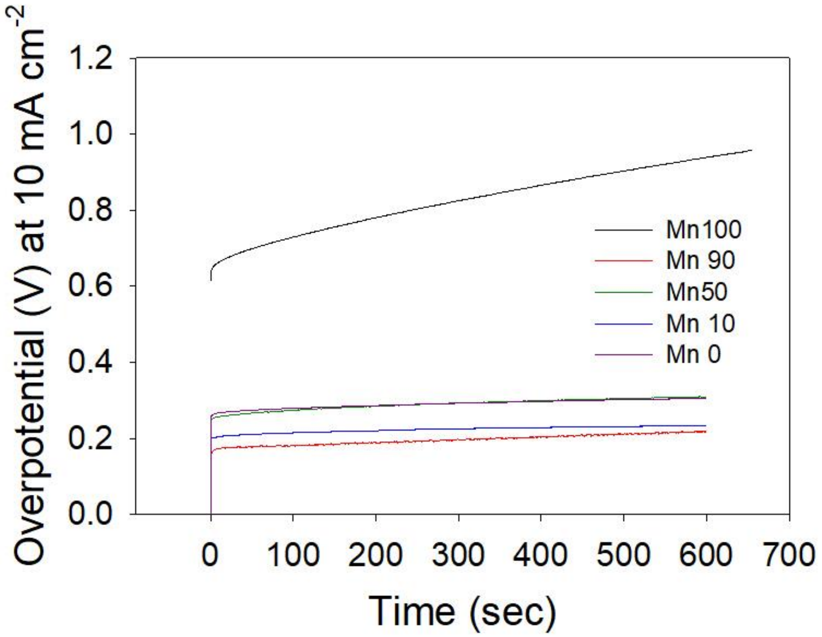

Figure S3. Chronoamperometry after correction by 90% iR drop. The data indicates that independently of iR correction, mixed Mn/Ru oxides show similar or better overpotentials than thermally deposited RuO_2 (Mn 0).

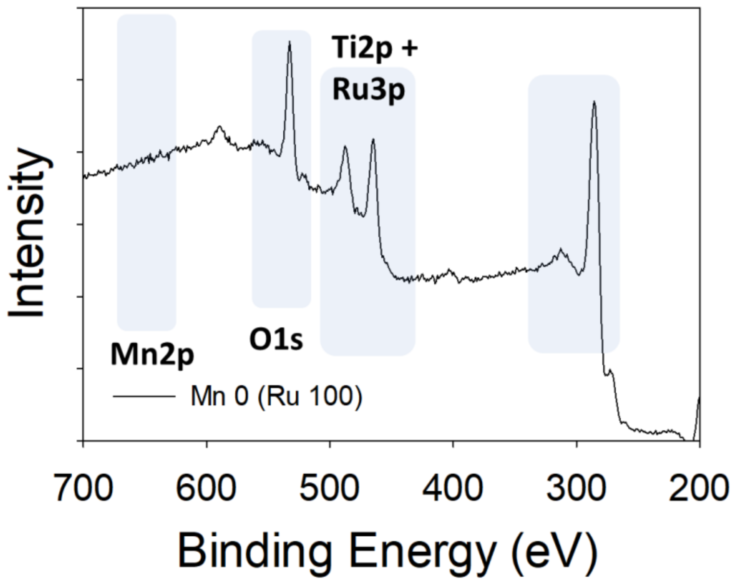

Figure S4. XPS survey of the Mn 0/ Ru 100 material.

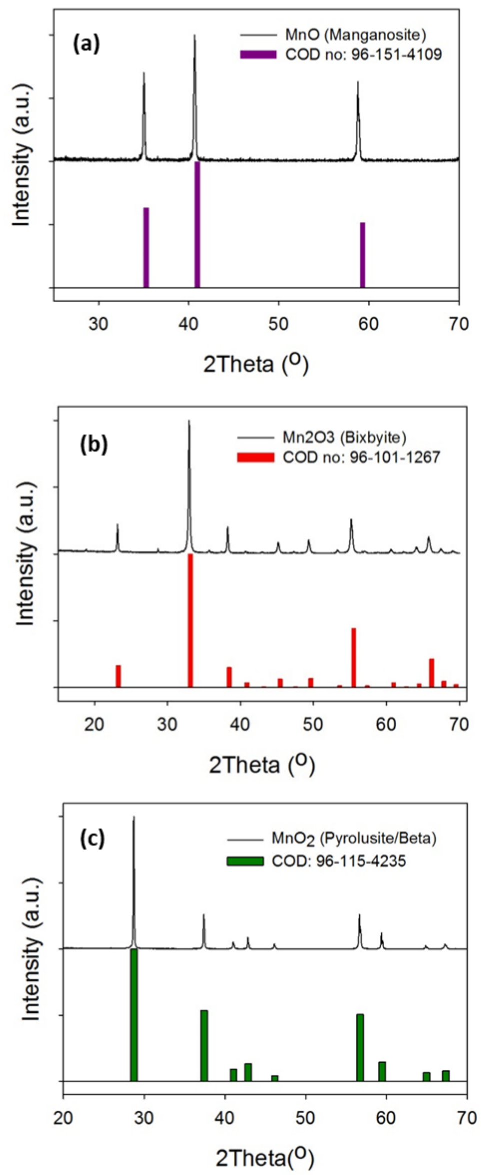

Figure S5. XRD pattern of Mn reference standards used **(a)** MnO **(b)** Mn_2O_3 and **(c)** MnO_2

Table S1. Commercial Mn oxide edge positions and oxidation states

Reference	Edge position (eV)	Mn oxidation state
**MnO**	6545.9	2
**Mn_2O_3**	6551.5	3
**MnO_2**	6555.1	4

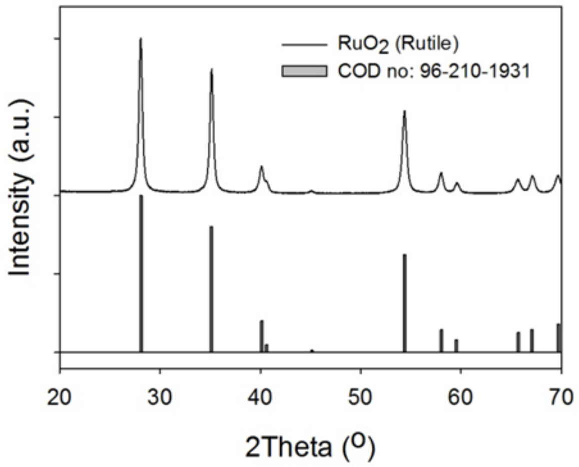

Figure S6. XRD patterns of RuO_2 reference used in the XAS measurements

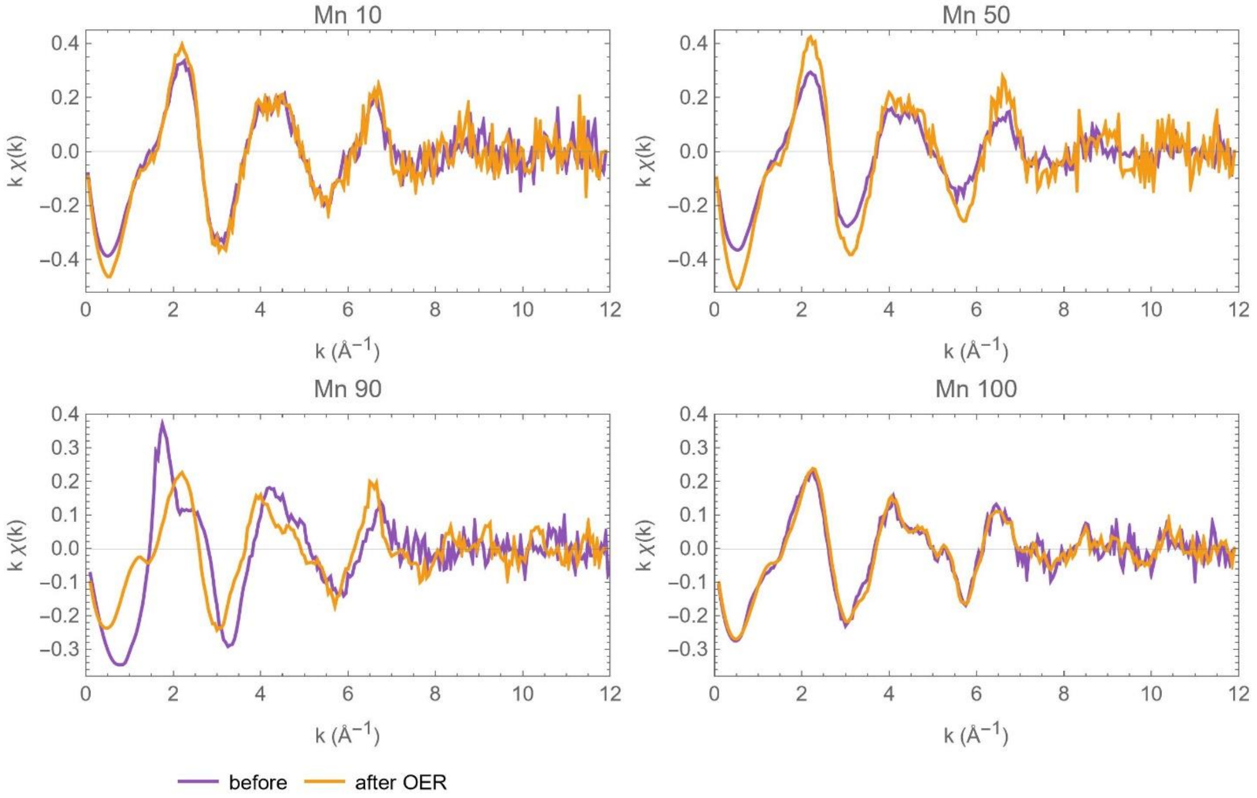

Figure S7. Comparison of EXAFS spectra of Mn 10-100 materials obtained at the Mn K-edge before and after OER at 10 mA cm^{-2}.

Table S2. Mn-O scattering paths used for the best fit of the first coordination shell of Mn/Ru electrodes at the Mn-edge before undergoing OER. Common values of S_0^2 = 0.65, obtained following calculations on standards, and of E_0 = -4 were used; parameters reported without an error were fixes.

**Sample**	**R (Å)**	**N**	**σ^2 (Å^2)**	**χ^2_{red}**
Mn 10	1.89(0.01)	4.6(0.4)	0.002(0.001)	2.8
Mn 50	1.89(0.01)	3.8(0.4)	0.002(0.002)	8.3
Mn 90	1.85(0.01)	3.4(0.3)	0.002	3.5
Mn 100	1.89(0.01)	2.4(0.2)	0.002	4.6

Table S3. Mn-O scattering paths used for the best fit of the first coordination shell of Mn/Ru electrodes at the Mn-edge after undergoing OER. Common values of S_0^2 = 0.65, obtained following calculations on standards, and of E_0 = -4 were used; parameters reported without an error were fixed.

**Sample**	**R (Å)**	**N**	**σ^2 (Å^2)**	**χ^2_{red}**
Mn 10	1.89(0.01)	4.9(0.3)	0.002	4.1
Mn 50	1.88(0.01)	5.4(0.5)	0.002	8.3
Mn 90	1.90(0.01)	3.1(0.3)	0.002	37
Mn 100	1.88(0.01)	2.7(0.2)	0.002	24

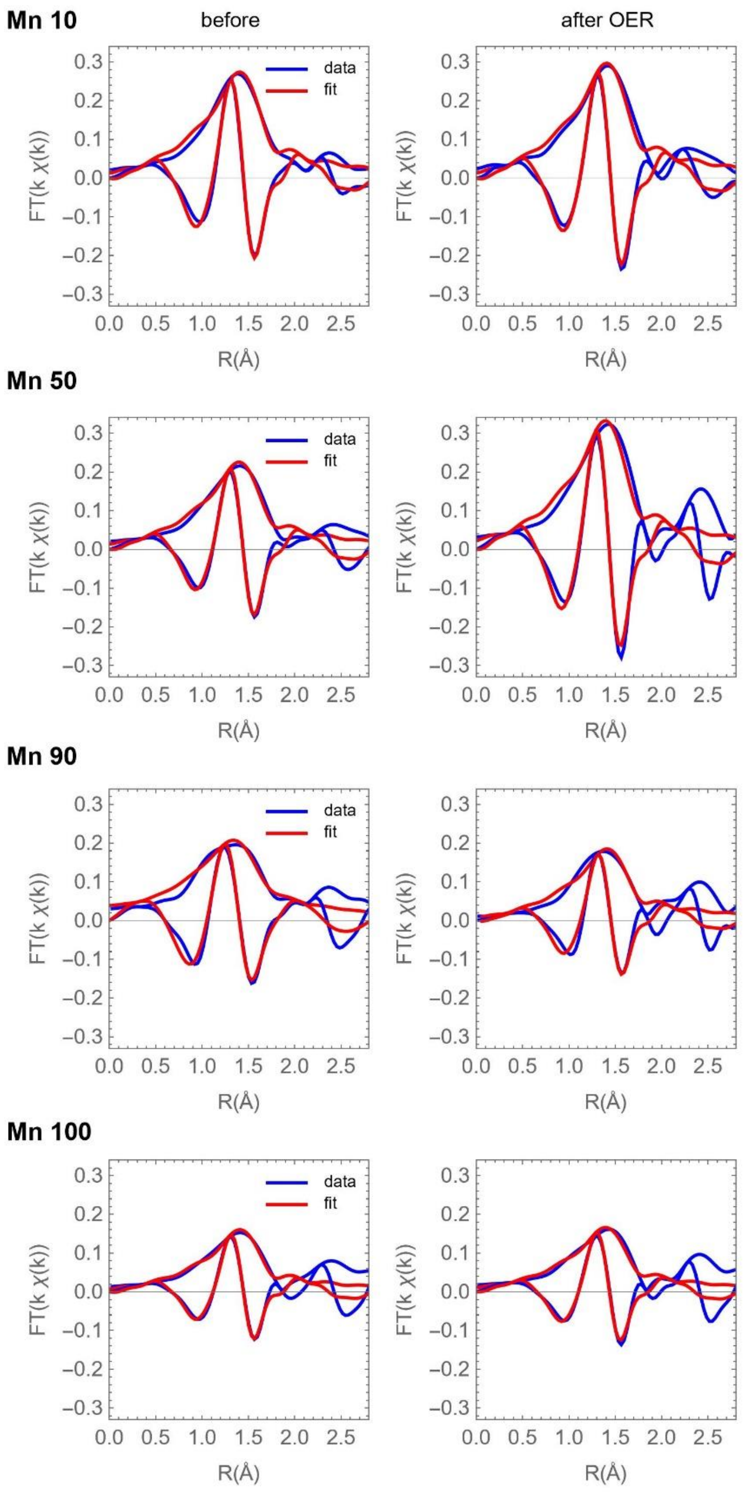

Figure S8. Comparison of spectra and best fits of the first coordination shell of Mn 10-100 materials obtained at the Mn K-edge; fitted spectra correspond to those shown in Figure S7. Both amplitude and imaginary part are shown; parameter results from these fits are shown in Tables S2-S3.

Table S4. Ru Edge positions of the Mn 100 – Mn10 materials in NaOH.

Material	In NaOH	reference
	Edge position (eV)	
**Mn 90**	22129.6	This work
**Mn 50**	22128.1	This work
**Mn 10**	22128.6	This work
**Mn 0**	22129.2	This work
**RuO_2 ref**	22128.9	This work

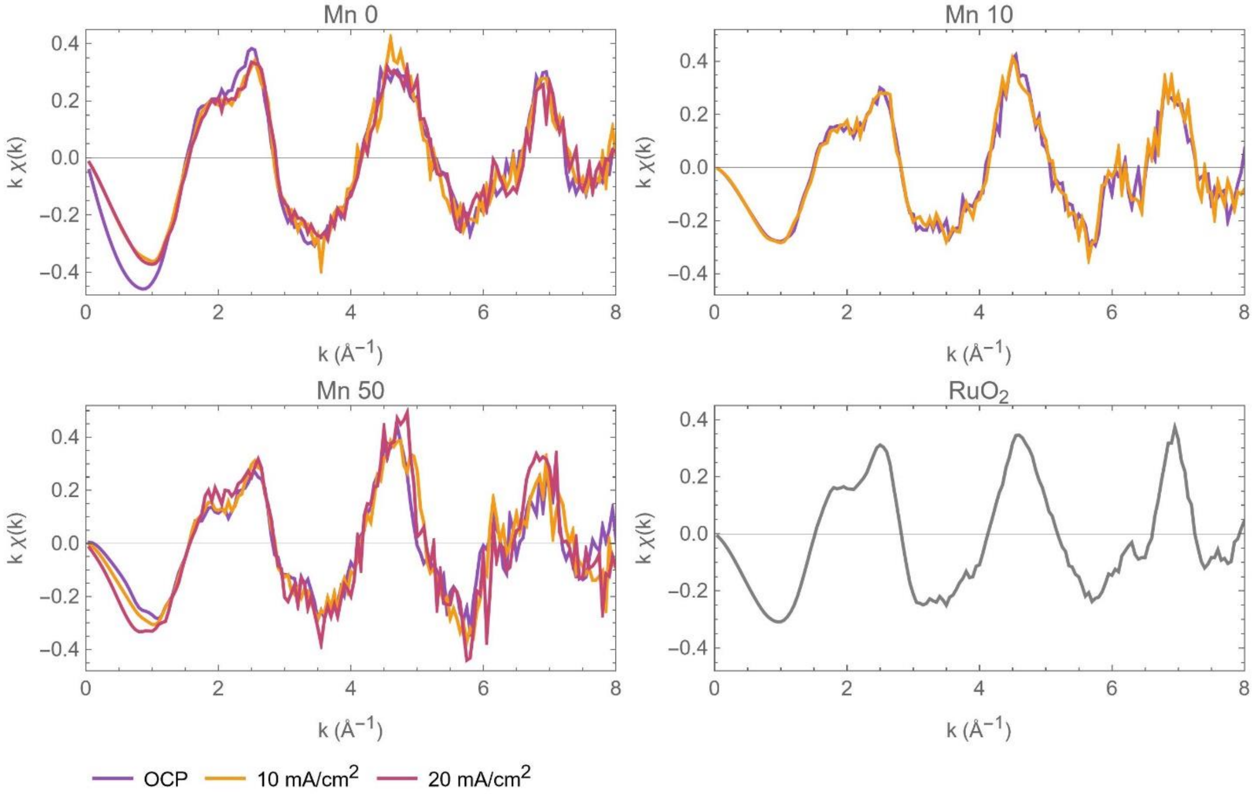

Figure S9. EXAFS spectra of Mn 10, 50 and Ru 100 (Mn 0) materials obtained at the Ru K-edge, measured at open circuit (OCP), during OER at 10 mA/cm^2, and at 20 mA/cm^2 for Mn 50 and Mn 0 (Ru 100). The EXAFS spectrum of the reference RuO_2 compound is also shown for comparison.

Table S5. Ru-O scattering paths used for best fits of the first coordination shell at the Ru-edge spectra at OCP and during OER at 10 mA/cm^2. Common values of S_0^2 = 0.75, obtained following calculations on standards, and of E_0 = 4.6 were used; parameters reported without an error were fixed.

**Sample**	**R (Å)**	**N**	**σ^2 (Å^2)**	**χ^2_{red}**
Mn 0 (Ru 100)	1.96(0.01)	6.3(0.6)	0.0026	34
Mn 0 at 10 mA/cm^2	1.95(0.01)	6.4(0.5)	0.0026	14
Mn 0 at 20 mA/cm^2	1.95(0.01)	5.8(0.4)	0.0026	8
Mn 10	1.98(0.01)	6.4(0.6)	0.0026	20
Mn 10 at 10 mA/cm^2	1.97(0.01)	6.5(0.8)	0.0026	27
Mn 50	1.97(0.01)	6.1(0.7)	0.0026	19
Mn 50 at 10 mA/cm^2	1.97(0.01)	7.1(1.0)	0.0026	26
Mn 50 at 20 mA/cm^2	1.96(0.01)	8.0(1.1)	0.0026	23

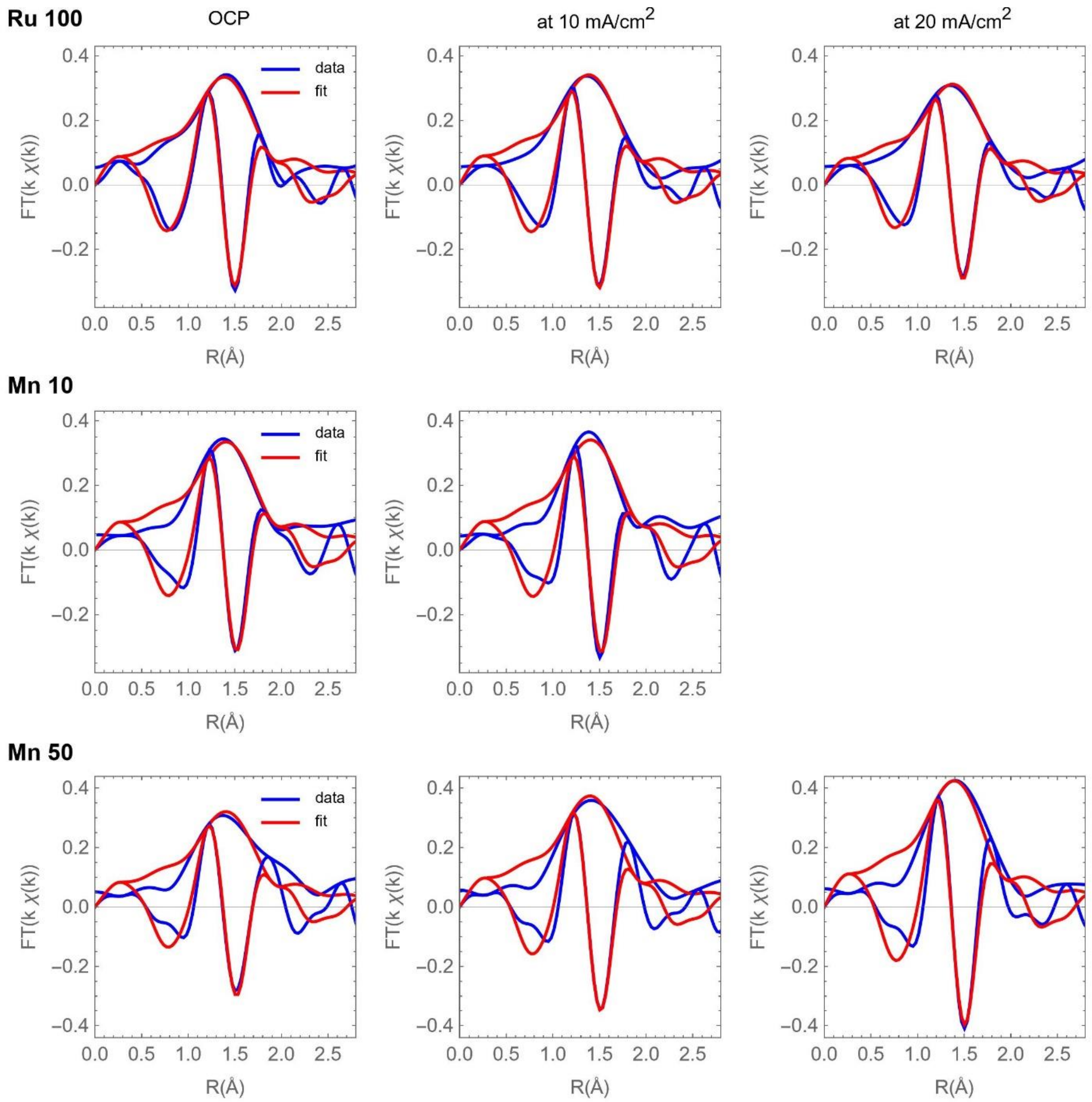

Figure S10. Comparison of spectra and best fits of the first coordination shell of Mn 0 (Ru 100), Mn 10 and Mn 50 materials obtained at the Ru K-edge at OCP (left), at 10 mA/cm^2 (center) and, where applicable, at 20 mA/cm^2 (right). Fitted spectra correspond to those shown in Figure S10; both amplitude and imaginary part are shown, while parameter results from these fits are shown in Tables S6.

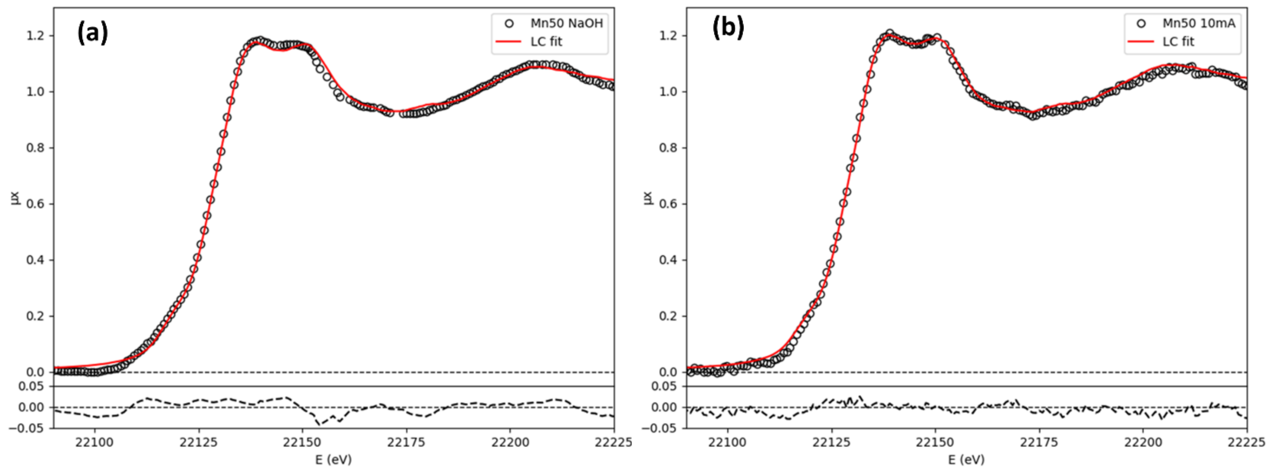

Figure S11. Linear combination (LC) fits of the Mn 50 sample **(a)** in NaOH and **(b)** at 10 mA cm^{-2}

Table S6. LC fit results of Mn 50 in NaOH and at 10 mA cm^{-2}; relative error of 10%.

	**Mn 50 in NaOH**	**Mn 50 in NaOH, 10mA**
**RuO_2**	81 %	89%
**Ru (metal)**	19 %	11%
**R-factor**	0.2%	0.2%

References

(1) Kibsgaard, J.; Chen, Z.; Reinecke, B. N.; Jaramillo, T. F. Engineering the surface structure of MoS2 to preferentially expose active edge sites for electrocatalysis. *Nat. Mater.* **2012**, *11*, 963-969. DOI: 10.1038/nmat3439.
